# Unveiling the importance of the C-terminus in the sugar acid dehydratase of the IlvD/EDD superfamily

**DOI:** 10.1007/s00253-024-13270-8

**Published:** 2024-08-10

**Authors:** Yaxin Ren, Elias Vettenranta, Leena Penttinen, Martina Blomster Andberg, Anu Koivula, Juha Rouvinen, Nina Hakulinen

**Affiliations:** 1 Department of Chemistry, University of Eastern Finland, Joensuu Campus, PO BOX 111, 80101 Joensuu, Finland; 2 VTT Technical Research Centre of Finland Ltd, Espoo, Finland

**Keywords:** L-Arabinonate dehydratase, IlvD/EDD superfamily, Dihydroxy acid dehydratase, Iron–sulfur cluster, Non-phosphorylative oxidative pathways

## Abstract

**Abstract:**

Microbial non-phosphorylative oxidative pathways present promising potential in the biosynthesis of platform chemicals from the hemicellulosic fraction of lignocellulose. An L-arabinonate dehydratase from *Rhizobium leguminosarum* bv. *trifolii* catalyzes the rate-limiting step in the non-phosphorylative oxidative pathways, that is, converts sugar acid to 2-dehydro-3-deoxy sugar acid. We have shown earlier that the enzyme forms a dimer of dimers, in which the C-terminal histidine residue from one monomer participates in the formation of the active site of an adjacent monomer. The histidine appears to be conserved across the sequences of sugar acid dehydratases. To study the role of the C-terminus, five variants (H579A, H579F, H579L, H579Q, and H579W) were produced. All variants showed decreased activity for the tested sugar acid substrates, except the variant H579L on D-fuconate, which showed about 20% increase in activity. The reaction kinetic data showed that the substrate preference was slightly modified in H579L compared to the wild-type enzyme, demonstrating that the alternation of the substrate preference of sugar acid dehydratases is possible. In addition, a crystal structure of H579L was determined at 2.4 Å with a product analog 2-oxobutyrate. This is the first enzyme-ligand complex structure from an IlvD/EDD superfamily enzyme. The binding of 2-oxobutyrate suggests how the substrate would bind into the active site in the orientation, which could lead to the dehydration reaction.

**Key points:**

•* Mutation of the last histidine at the C-terminus changed the catalytic activity of L-arabinonate dehydratase from R. leguminosarum bv. trifolii against various C5/C6 sugar acids.*

•* The variant H579L of L-arabinonate dehydratase showed an alteration of substrate preferences compared with the wild type.*

•* The first enzyme-ligand complex crystal structure of an IlvD/EDD superfamily enzyme was solved.*

**Graphical Abstract:**

**Figure:**
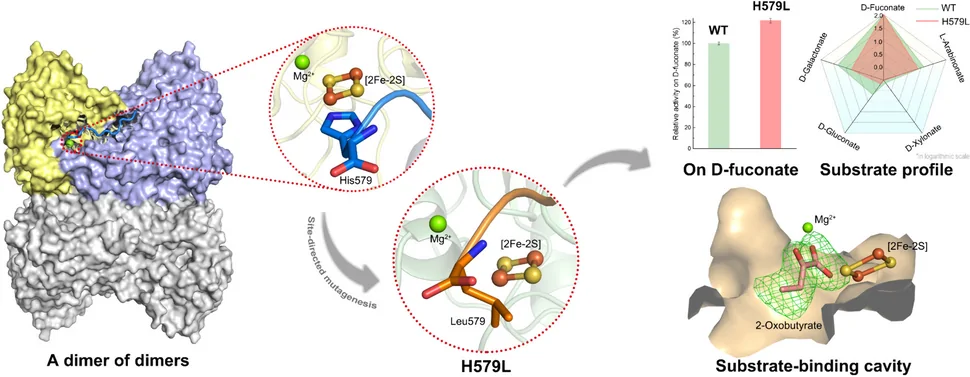

**Supplementary Information:**

The online version contains supplementary material available at 10.1007/s00253-024-13270-8.

## Introduction

Pentose sugars from hemicellulose are promising feedstock for producing various platform chemicals, polymer precursors, and biofuels (Büchert et al. 1989; Menon and Rao 2012). Microbial non-phosphorylative oxidative pathways have recently gained much attention because of the advantages of having fewer conversion steps and less complex regulation than other pathways. Pentose sugars can be converted into α-ketoglutarate by the Weimberg pathway (Weimberg 1961), glycolaldehyde and pyruvate by the Dahms pathway (Dahms 1974), or glycolate and pyruvate by the pathway discovered by Watanabe et al. (2019). In all these pathways, the rate-limiting step is the third step of the reaction—dehydration of the sugar acid to 2-dehydro-3-deoxy sugar acid, catalyzed by sugar acid dehydratases (Tai et al. 2016). Understanding the structure and function of the sugar acid dehydratases enhances their usage for industrial applications, thereby boosting the possibility of employing microbial non-phosphorylative oxidative pathways in pentose bioconversion (Ren et al. 2022).

Sugar acid dehydratases generally belong to either the Mg-dependent enolase superfamily or the FeS-dependent IlvD/EDD (isoleucine, leucine, valine dehydratase/Entner-Doudoroff dehydratase) superfamily. The name of the IlvD/EDD superfamily derives originally from two dehydratases: a dihydroxy-acid dehydratase (IlvD, EC 4.2.1.9), which is involved in the synthesis of branched-chain amino acids isoleucine, leucine and valine (Myers 1961), and a phosphogluconate dehydratase (EDD, EC 4.2.1.12), which is involved in the Entner-Doudoroff (ED) pathway, in which 6-phospho-D-gluconate is converted to 6-phospho-2-dehydro-3-deoxy-D-gluconate (Meloche and Wood 1964). Based on the substrate specificity on different dihydroxy acids and the evolutionary relationship of enzymes, Melse et al. (2022) recently proposed classifying [2Fe-2S]-dependent IlvD enzymes into three classes: (1) sugar acid dehydratases (SADHTs), which are active on five to six carbon (C5/C6) sugar acids, such as, D-xylonate, L-arabinonate, and D-gluconate; (2) branched chain acid dehydratases (BCADHTs), which prefer branched dihydroxy acids, such as (R)-2,3-dihydroxy isovalerate, as substrates; and (3) promiscuous acid dehydratases, which have comparable activity on both branched and open-chain dihydroxy acid substrates.

Currently, there are 14 structures of eight orthologous dehydratases from the IlvD/EDD superfamily released in the Protein Data Bank (PDB), but there are no any crystal structures complexed with an inhibitor, a substrate/product, or their analogs. The overall structure of Ilv/ED dehydratases is composed of an N-terminal αβα-sandwich domain and a C-terminal β-barrel domain (Rahman et al. 2017). Their quaternary structure is either a homodimer or a homotetramer, which can also be regarded as a dimer of dimers. The active site is in the cavity between the two domains of a monomer and in the interface between the two monomers in the dimer. Therefore, the dimerization of Ilv/ED dehydratase is required to form an active enzyme with two active sites per dimer. Each active site contains a [2Fe-2S] cluster and a magnesium ion.

An L-arabinonate dehydratase from *Rhizobium leguminosarum* bv. *trifolii* (hereafter referred to as *Rl*ArDHT) belongs to the sugar acid dehydratase subfamily. A quaternary structure of *Rl*ArDHT is a homotetramer (Rahman et al. 2017), and the active site is formed in the dimeric interface in a manner in which an N-terminal α-helix (particularly residues Arg22 − Trp30) and a C-terminus of one monomeric unit contribute to the formation of the active site of the partnering monomer. In this study, the role of C-terminal histidine in *Rl*ArDHT was studied using site-directed mutagenesis. Five C-terminal variants were designed, and their relative activities against various C5/C6 substrates were tested. The kinetic parameters (*K*_m_ and *k*_cat_) of the H588L variant were further determined on various substrates, and a crystal structure of H588L co-crystallized with 2-oxobutyrate was solved at 2.4 Å resolution.

## Materials and methods

### Strain, vector, and substrates

*Escherichia coli* BL21(DE3) competent cells for heterologous target gene expression were purchased from Thermo Scientific (Waltham, MA, USA). The plasmid pBAT4 was used as the expression vector (Peränen et al. 1996). All substrates used in this study were commercially available. L-threonate was purchased from United States Pharmacopeia (Rockville, MD, USA). L-arabinonate was obtained from Biosynth (Compton, UK), and D-xylonate was sourced from Santa Cruz Biotechnology (Paso Robles, CA, USA). D-fuconate, D-galactonate, and D-gluconate were all purchased from Sigma-Aldrich (St. Louis, MI, USA).

### Gene cloning and site-directed mutagenesis

The encoding gene of L-arabinonate dehydratase from *R. leguminosarum* bv. *trifolii* (*araD*, GenBank code: KT260159) was synthesized with codon optimization based on *E. coli* codon preference by GeneArt (Regensburg, Germany). A Strep-tag II consisting of eight amino acids (Trp, Ser, His, Pro, Gln, Phe, Glu, and Lys) was added at the N-terminus of *Rl*ArDHT. The tagged gene was cloned into the plasmid pBAT4 using *EcoR*I and *Not*I restriction sites, and the recombinant plasmid pBAT4-ArDHT was used in the *E. coli* BL21(DE3) expression system.

The site-directed mutagenesis of the gene *araD* was performed by GenScript’s Site-Directed Mutagenesis Services (Piscataway, NJ, USA), with the recombinant plasmid pBAT4-ArDHT as the template. The following five C-terminal variants of *Rl*ArDHT were constructed: (1) *Rl*ArDHT-H579A, (2) *Rl*ArDHT-H579F, (3) *Rl*ArDHT-H579L, (4) *Rl*ArDHT-H579Q, and (5) *Rl*Ar*DHT*-H579W. The mutated recombinant plasmids were introduced into *E. coli* BL21(DE3) for protein expression.

### Protein expression and purification

The recombinant plasmids were transformed into *E. coli* BL21(DE3) competent cells according to the manufacturer’s instructions. Positive transformants were selected by their resistance to ampicillin for further shake flask fermentation. The positive transformant was first inoculated into the Lysogenic broth (LB) overnight for growth at 37 °C (200 rpm) to prepare the seed medium. Then, the seed medium was transferred into 500 mL of LB medium in 2 L conical flasks with a 1% (v/v) inoculum and cultivated at 37 °C (200 rpm) until the A_600_ = 0.5–0.8 was reached. Next, 1 mM of isopropyl-β-D-thiogalactopyranoside was added to induce the expression of the protein, and the culture was further grown at 37 °C (200 rpm) for 4 h. All mediums were supplemented with 100 ug mL^−1^ ampicillin.

After growth, the cells were harvested by centrifugation for 30 min at 4 °C (4000 × g) and resuspended in ice-cold lysis buffer containing 50 mM Tris–HCl, 150 mM NaCl, 5 mM MgCl_2_, 1 mM dithiothreitol (DTT), and ethylenediaminetetraacetic acid (EDTA)-free protease inhibitors (Roche, Basel, Switzerland) at pH 8.0. The cells were distributed by sonification on ice and centrifuged for 30 min at 4 °C (12,000 × g) to remove the cell debris and undissolved materials. The cell-free extract was then loaded on a Strep-Tactin Superflow column (GE Healthcare, Stockholm, Sweden) equilibrated by the equilibrium buffer (50 mM Tris–HCl, 150 mM NaCl, and 5 mM MgCl_2_ at pH 8.0). The bound proteins were eluted with 2.5 mM D-desthiobiotin (Sigma-Aldrich, Darmstadt, Germany) in the equilibrium buffer. Fractions containing the target protein were pooled and assayed in sodium dodecyl sulfate–polyacrylamide gel electrophoresis (SDS-PAGE). Next, the buffer of the purified proteins was exchanged into 50 mM Tris–HCl and 5 mM MgCl_2_ at pH 7.5 by gel filtration with a Superdex 200 gel filtration column (GE Healthcare, Stockholm, Sweden). The proteins were concentrated using Vivaspin 2 (MWCO 10000 Da, GE Healthcare, Stockholm, Sweden). The protein concentration of the purified enzymes was calculated from A_280_ measured using Eppendorf Biophotometer (Hamburg, Germany) with a theoretical extinction coefficient of 76,890 M^−1^ cm^−1^ calculated by ProtParam (http://au.expasy. org/tools/protparam.html).

### UV–vis spectroscopy

The UV–vis spectrum of wild-type and C-terminal variants (2.5 mg mL^−1^ in 50 mM Tris–HCl and 5 mM MgCl_2_ at pH 7.5) was measured by using a Lambda 900 UV/VIS/NIR spectrophotometer (PerkinElmer Inc, Waltham, MA, USA) from 300 to 700 nm, at 21 °C. The same buffer in the absence of protein was used as a blank.

### Measurement of enzyme activity and kinetic constants

Dehydratase activity was assayed at 30 °C in 50 mM Tris–HCl buffer and 10 mM MgCl_2_ at pH 8.0. The reaction mixture consisted of 400 µl of 20 mM sugar acid and 400 µl of the appropriate diluted purified enzyme solution. Six time points were set for each measurement, and after every one-sixth of the reaction time, 100 µl of the reaction mixture was removed, and the reaction was terminated by adding 12% (w/v) trichloroacetic acid. The formed product, 2-dehydro-3-deoxy-pentonate or 2-dehydro-3-deoxy-hexonate, was determined using thiobarbituric acid (TBA) assay (ε = 67,800 M^−1^ cm^−1^ at 549 nm) (Buchanan et al. 1999). The rate of reaction was calculated by linear regression analysis using Microsoft Excel version 16.66.1 (Redmond, WA, USA).

The kinetic analysis of *Rl*ArDHT wild-type enzyme and the variant H579L was carried out using 0.15–10 mM D-fuconate, L-arabinonate, D-galactonate, D-xylonate, and D-gluconate at 30 °C in 50 mM Tris–HCl buffer and 10 mM MgCl_2_ at pH 8.0. The kinetic constants were determined using GraphPad Prism version 5.01 (La Jolla, CA, USA) with the Michaelis Menten model. All reactions were performed in triplicate.

### Crystallization and data collection

The H579L variant was co-crystallized with the product analog 2-oxobutyrate (Sigma-Aldrich, St. Louis, MI, USA) using the hanging-drop vapor diffusion method at 20 °C. Initially, small, serried, needle-like crystals of variant H579L were observed in the droplet, consisting of 2 µl of 3 mg ml^−1^ of purified protein solution (in buffer 50 mM Tris–HCl, 5 mM MgCl_2_, pH 7.5), 1 µl of 100 mM 2-oxobutyrate solution, 0.5 µl of 100 mM DTT, and 1.5 µl of crystallization solution (15% (w/v) PEG 10000, 200 mM ammonium acetate, and 100 mM Tris, pH 8.5). Better crystals were obtained using the streak-seeding method with a dog hair to transfer the nucleates into another pre-equilibrate droplet with 14% (w/v) PEG 10000, 200 mM ammonium acetate, and 100 mM Tris at pH 8.5. Crystals appeared in a droplet within two weeks. Glycerol 30% (v/v) was used as the cryoprotectant. The 2.4 Å data set was collected on beamline ID30B at the European Synchrotron Radiation Facility (ESRF) and processed using the XDS program package (Kabsch 2010). Detailed data collection statistics are shown in Table 1.

**Table 1:** Data collection and refinement statistics

PDB ID	9EVV
Data collection	
Space group	P2_1_ 2_1_ 2_1_
Cell dimension	
a, b, c (Å)	107.947, 148.211, 165.025
α, β, γ (°)	90, 90, 90
Resolution (Å)	48.48–2.44 (2.53–2.44)
* R*_meas_ (%)	34.2 (212.6)
* I/σ* (*I*)	5.66 (0.96)
* CC*_1/2_	0.986 (0.45)
Total reflections	675,826 (62,867)
Unique reflections	98,251 (9397)
Completeness (%)	99.6 (96.9)
Multiplicity	6.9 (6.7)
Refinement statistics	
Reflection used in refinement	98,218 (9396)
Reflection used for R-free	1999 (191)
Molecules per asymmetric unit	4
R-work	0.1851 (0.2860)
R-free	0.2367 (0.3276)
Number of non-hydrogen atoms	17,860
Protein	17,402
Water	431
Fe/S	8/8
Mg	4
2-Oxobutyrate	7
Average *B-factor* (Å^2^)	42.54
Protein	42.58
Water	40.84
Fe/S	41.09/40.48
Mg	38.98
2-Oxobutyrate	49.50
R.m.s. deviations	
Bond lengths (Å)	0.008
Bond angles (°)	0.98
Ramachandran plot (%)	
Favored	96.32
Outliers	0.26

Values in parentheses are for the highest-resolution shell

*R.m.s.* root mean square

### Structure determination and refinement

A crystal structure of the *Rl*ArDHT variant H579L complexed with 2-oxobutyrate at 2.4 Å was determined by molecular replacement using Phaser from the PHENIX software suite (McCoy et al. 2007), and the published holo-form structure of wild-type *Rl*ArDHT (PDB ID: 5J84) was used as a template. Model building was done manually in COOT (Emsley et al. 2010) and PHENIX refine was used in the refinement of the model (Afonine et al. 2012). Water molecules were initially added using the “Update Water” option and then evaluated and corrected manually. The unbiased F_o_-F_c_ omit map and additional polder omit map (Liebschner et al. 2017) were used to locate 2-oxobutyrate, and the geometric restraints of 2-oxobutyrate were calculated using eLBOW (Moriarty et al. 2009). Detailed refinement statistics are shown in Table 1.

## Results

### C-terminus in the sugar acid dehydratase subfamily

**Fig. 1:**
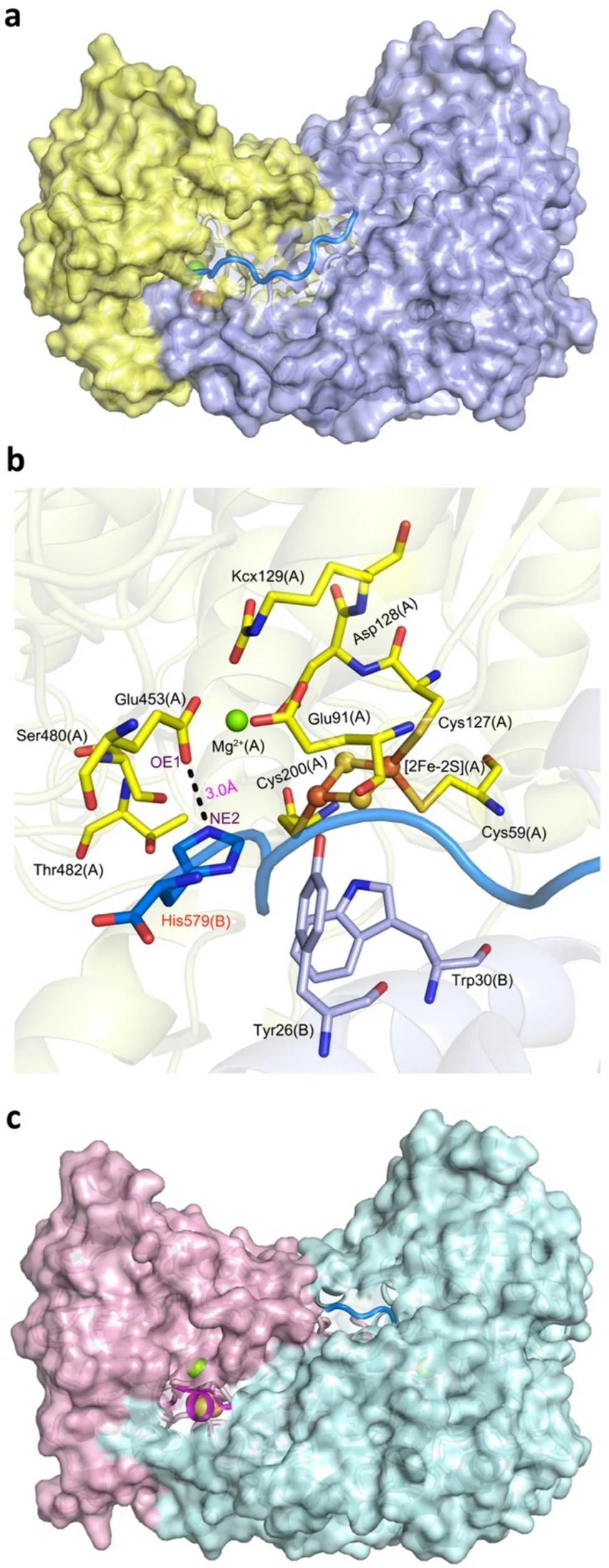
The dimeric unit (**a**) and the substrate-binding cavity (**b**) of L-arabinonate dehydratase from *Rhizobium leguminosarum* bv. *trifolii* (PDB ID: 5J84). Chain A is shown in yellow, chain B is in purple-blue, and the long C-terminal tail from chain B is in marine. **c** The dimeric unit of dihydroxy acid dehydratase from *Mycobacterium tuberculosis* (PDB ID: 6OVT). Chain A is shown in pink and chain B in turquoise, the short C-terminal tail from chain B is shown in marine, and the short extra α-helix is shown in magenta

The crystal structures of the wild-type *Rl*ArDHT have been solved with and without the iron–sulfur cluster as apo form (PDB ID: 5J83) and holo form (PDB ID: 5j84), respectively (Rahman et al. 2017). These structures revealed a long random coil C-terminal region (residues 567 − 579), in which the last five residues interact with the adjacent monomer. Dimerization pairs the two monomers in such a way that the C-terminal tails of both monomers bind to the active site of the partnering monomer (Fig. 1a). The final quaternary structure of *Rl*ArDHT is a homotetramer, which can be seen as a dimer of dimers.

The last residue of the C-terminal tail, His579, participates in the formation of the substrate-binding cavity of the neighboring monomer (Fig. 1b). The imidazole group of His579 (NE2 atom) is 3.0 Å from the carboxylate group of Glu453 (OE1 atom), and a salt bridge is able to form between each other. Glu453 is one of the residues responsible for coordinating Mg^2+^.

**Fig. 2:**
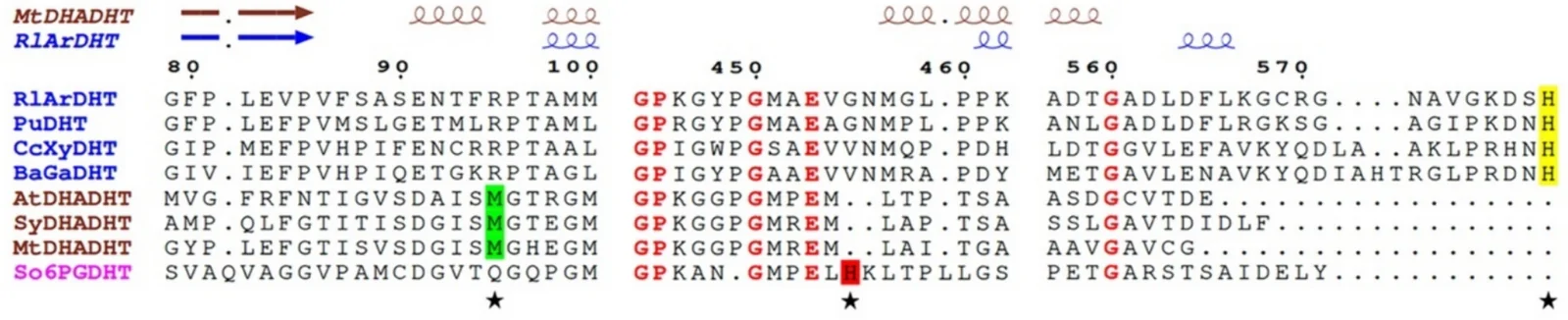
Multiple sequence alignment of all IlvD/EDD superfamily dehydratases with known crystal structures. *Rl*ArDHT is an L-arabinonate dehydratase from *Rhizobium leguminosarum* bv. *trifolii* (PDB ID: 5J84); *Pu*DHT is a sugar acid dehydratase from *Paralcaligenes ureilyticus* (PDB ID: 8EPZ); *Cc*XyDHT is a D-xylonate dehydratase from *Caulobacter crescentus* (PDB ID: 5OYN); *Ba*GaDHT is a galactonate dehydratase from *Brucella abortus* 2308 (PDB ID: 7M3K); *At*DHADHT is a dihydroxy acid dehydratase from *Arabidopsis thaliana* (PDB ID: 5YM0); *Sy*DHADHT is a dihydroxy acid dehydratase from *Synechocystis* sp. PCC 6803 (PDB ID: 6NTE); *Mt*DHADHT is a dihydroxy acid dehydratase from *Mycobacterium tuberculosis* (PDB ID: 6OVT); and *So*6PGDHT is a 6-phosphogluconate dehydratase from *Shewanella oneidensis* MR-1 (PDB ID: 2GP4). The names of sugar acid dehydratases are in blue, and those of branched chain acid dehydratases are in brown. The conserved C-terminal histidine residues of sugar acid dehydratases are marked in yellow, the conserved methionine residues in branched chain acid dehydratases are marked in green, and the His488 residue in *So*6PGDHT is marked in red. These are also marked with a black star at the bottom of the sequences. Fully conserved residues are represented in red. The numbers above the sequences indicate the residue number of *Rl*ArDHT. Secondary structure elements of *Mt*DHADHT and *Rl*ArDHT are also shown

A similar structural role of the C-terminal histidine has also been observed in another pentonate dehydratase, a D-xylonate dehydratase from *Caulobacter crescentus* (PDB ID: 5OYN) (Rahman et al. 2018), and in a very recently solved sugar acid dehydratase from *Paralcaligenes ureilyticus* (PDB ID: 8EPZ, 8EJ0) as well (Bayaraa et al. 2023a, b). The last histidine residue is fully conserved among SADHT subfamily members in the IlvD/EDD superfamily (Fig. 2 and Supplemental Fig. S1). The sequence alignment suggests that a galactonate dehydratase from *Brucella abortus* 2308 (PDB ID: 7M3K) also belongs to this SADHT subfamily, although its C-terminal residues are missing in the published crystal structure (Abendroth et al. 2021).

In contrast to SADHTs, BCADHTs have different C-terminal arrangements, and they do not have protruding C-terminal histidines (Bashiri et al. 2019). The crystal structures of the following BCADHTs have been solved: a dihydroxy acid dehydratase from *Arabidopsis thaliana* (PDB ID: 5YM0, 5ZE4) and its variant V178W (PDB ID: 8HS0) (Yan et al. 2018; Zang et al. 2018; Zhou et al. 2024), a dihydroxy acid dehydratase from *Synechocystis* sp. PCC 6803 (PDB ID: 6NTE) (Zhang et al. 2020), and a dihydroxy acid dehydratase from *Mycobacterium tuberculosis* (PDB ID: 6OVT). The C-terminus of BCADHTs is shorter than that of SADHTs, and their orientation are not toward the active site of the adjacent monomer (Fig. 1c and Supplemental Fig. S2). Furthermore, the last residue is not conserved among BCADHTs (Fig. 2 and Supplemental Fig. S1). However, all the above-mentioned BCADHTs have a short extra α-helix (Fig. 1c) with the fully conserved methionine residue (Met100 in *Mt*DHAT) in the atomic position of the observed C-terminal histidine in SADHTs (Fig. 2 and Supplemental Fig. S2). A 6-phosphogluconate dehydratase from *Shewanella oneidensis* MR-1 (PDB ID: 2GP4), which is involved in the ED pathway, seems to form its own distinct subfamily (Schormann and Symersky 2006; Melse et al. 2022), but it also has a non-terminal His488 residue in the same place as the C-terminal histidine in SADHTs.

### Engineered C-terminal variants

Five variants (H579A, H579F, H579L, H579Q, and H579W) were generated to investigate the role of the C-terminal residue in *Rl*ArDHT. The mutated and wild-type recombinant *Rl*ArDHT were expressed in *E. coli* BL21 [DE3]) and the purified proteins were analyzed using SDS-PAGE (Supplemental Fig. S3). The amounts of the purified protein obtained for the variants and the wild type were similar, implying that the introduction of the mutations did not affect the expression level of the protein. All purified enzymes had a brown color because of the presence of an iron–sulfur cluster. The UV–vis spectra of all variants and wild-type enzymes showed the same peaks or shoulders appearing at around 325 nm, 385 nm, 440–480 nm, and 580 nm (Supplemental Fig. S4). This suggests that the substitutions of the C-terminal His residue did not influence the binding of the iron–sulfur cluster.

The activities of the five variants on various sugar acids (L-arabinonate, D-xylonate, D-gluconate, D-fuconate, and L-threonate) were measured and compared with those of the wild-type enzyme (Table 2). With L-arabinonate as the substrate, all variants showed a clear decrease in activity. The specific activity on L-arabinonate of the *Rl*ArDHT wild-type enzyme was 9.1 µmol min^−1^ mg^−1^ at 30 °C and pH 8.0, whereas under the same conditions, the observed activities were 1.1, 1.0, 1.7, 1.2, and 0.03 µmol min^−1^ mg^−1^ for the H579A, H579F, H579L, H579Q, and H579W mutants, respectively. The activity of the H579W variant on L-arabinonate decreased the most dramatically, as the mutation of His to Trp resulted in an almost complete loss of activity. Variants H579A, H579F, and H579Q had a similar level of reduced activity on L-arabinonate. Among the variants, H579L maintained the highest activity on L-arabinonate, with about 80% activity lost compared with that of the wild-type enzyme.

**Table 2:**
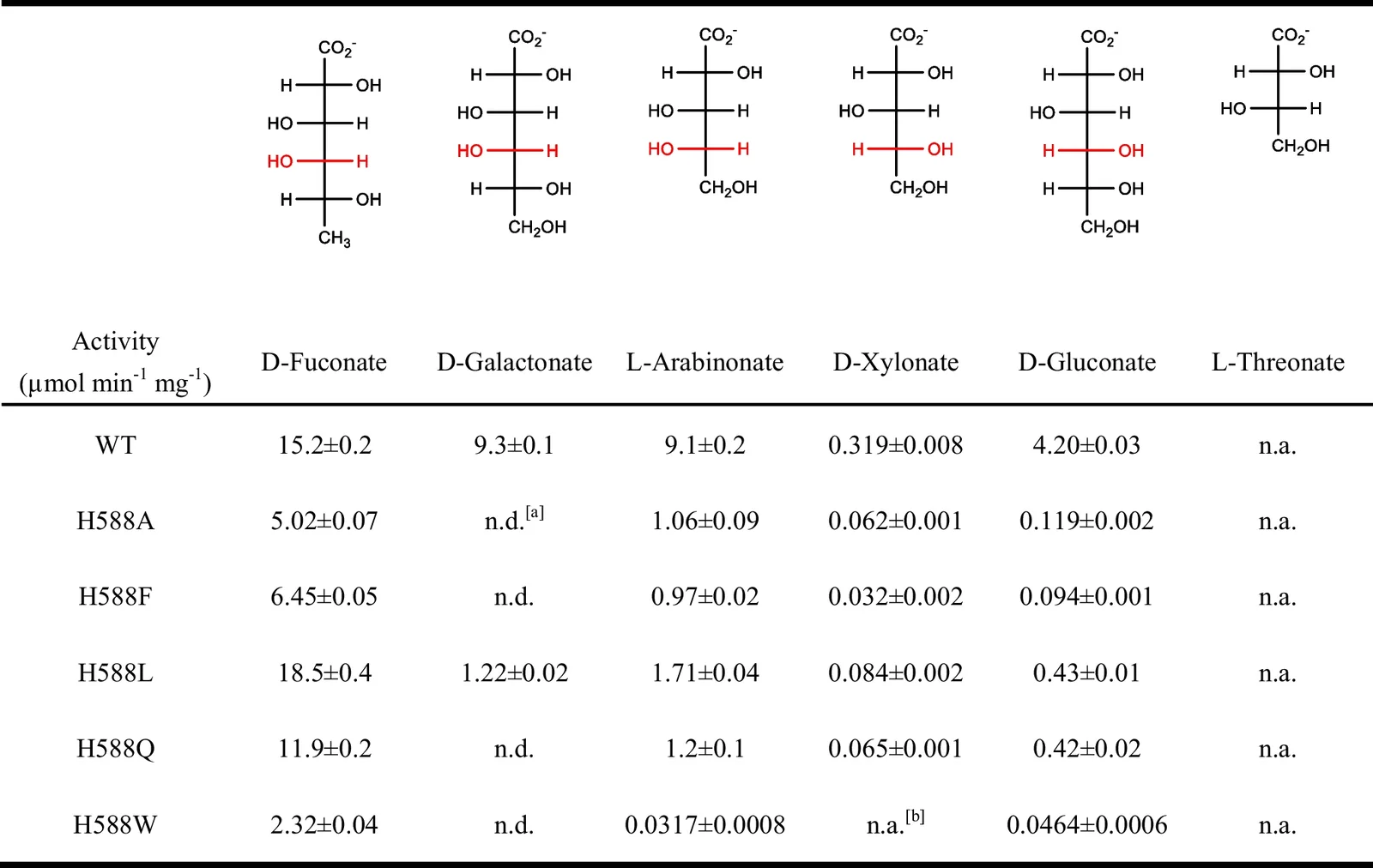
Specific activities of wild-type *Rl*ArDHT and its variants on various substrates measured at pH 8.0, 30 °C

^[a]^ n.d. means not determined; ^[b]^n.a. means the activity < 0.01 µmol min^−1^ mg^−1^

The hydroxyl group and the hydrogen atom at the C4 position are marked in red in the Fischer projections of the five substrates, as *Rl*ArDHT has a stereoselectivity for the S configuration at the C4 position of the substrate

The mutations of the last C-terminal residue made the weak activity of *Rl*ArDHT on D-xylonate even weaker. The specific activity of the wild-type enzyme on D-xylonate was only 0.3 µmol min^−1^ mg^−1^ at 30 °C and pH 8.0, and the activities of all variants were less than 0.08 µmol min^−1^ mg^−1^ (Table 2). The variants also showed a significant decrease in D-gluconate activity. The specific activity of the wild-type enzyme on D-gluconate was 4.2 µmol min^−1^ mg^−1^, and the specific activities of H579A, H579F, H579L, H579Q, and H579W were 0.12, 0.09, 0.43, 0.42, and 0.05 µmol min^−1^ mg^−1^, respectively.

Regarding D-fuconate, which is a 6-deoxy hexonate with the same configurations on positions C2, C3, and C4 as the L-arabinonate, the mutations on the last C-terminal His residue had an interesting effect on the activity. The specific activity of the wild-type enzyme on D-fuconate was 15.2 µmol min^−1^ mg^−1^ at 30 °C and pH 8.0. Variants H579A, H579F, H579Q, and H579W showed varying degrees of activity declines. The specific activity of H579A was 5.0 µmol min^−1^ mg^−1^, which is about one-third of the activity of the wild type. The specific activity of H579F was slightly higher than that of H579A, 6.4 µmol min^−1^ mg^−1^, and the specific activity of H579Q was 11.9 µmol min^−1^ mg^−1^. Variant H579W showed the largest decline in activity at 2.3 µmol min^−1^ mg^−1^. Contrary to the above-mentioned variants, H579L surprisingly had increased activity toward D-fuconate. The specific activity of H579L was 18.5 µmol min^−1^ mg^−1^, which was approximately 20% higher than that of the wild type.

We also tested the activity of the wild type and the variants on L-threonate, which is a C4 sugar acid compound. However, no activity of the wild type or the variants was detected on L-threonate at 30 °C and pH 8.0 using TBA assay.

### Kinetic analysis of the H579L variant

Because of the increased specific activity of the H579L variant on D-fuconate but not on L-arabinonate, we conducted further characterization of the catalytic parameters for H579L. The kinetic constants of the H579L variant and the wild-type enzyme on five substrates (L-arabinonate, D-xylonate, D-gluconate, D-fuconate, and D-galactonate) were determined at 30 °C, pH 8.0 (Table 3).

**Table 3:** Kinetic constants of the wild-type and variant H579L of *Rl*ArDHT on various substrates measured at pH 8.0, 30 °C

H579L			Substrate	WT		
*k*_cat_/*K*_m_ (mM^−1^ min^−1^)	*k*_cat_ (min^−1^)	*K*_m_ (mM)		*K*_m_ (mM)	*k*_cat_ (min^−1^)	*k*_cat_/*K*_m_ (mM^−1^ min^−1^)
120 ± 20	120 ± 5	1.0 ± 0.2	L-Arabinonate	1.2 ± 0.2	680 ± 30	570 ± 70
1570 ± 80	1290 ± 30	0.82 ± 0.06	D-Fuconate	0.24 ± 0.07	1050 ± 60	4400 ± 1100
95 ± 11	89 ± 4	0.90 ± 0.15	D-Galactonate	0.74 ± 0.15	690 ± 30	930 ± 140
7.5 ± 0.7	40 ± 3	5.4 ± 0.9	D-Gluconate	5.7 ± 1.5	390 ± 50	68 ± 9
5.3 ± 0.7	6.4 ± 0.2	1.2 ± 0.2	D-Xylonate	1.3 ± 0.3	24.8 ± 1.3	19 ± 4

For the dehydration of L-arabinonate, the *K*_m_ value of the H579L variant was almost fully comparable with that of the wild-type enzyme (1.0 mM vs. 1.2 mM), while the *k*_cat_ value of the variant was clearly lower (120 min^−1^ vs. 680 min^−1^). A similar phenomenon was also observed when the kinetic constants of the H579L variant and the wild-type enzyme on D-xylonate (*K*_m_ value of 1.2 mM and 1.3 mM; *k*_cat_ value of 6.4 min^−1^ and 24.8 min^−1^) and D-gluconate (*K*_m_ value of 5.4 mM and 5.7 mM; *k*_cat_ value of 40 min^−1^ and 390 min^−1^) were determined. These results indicate that the mutation of the His residue to Leu did not affect the affinity for L-arabinonate, D-xylonate, or D-gluconate. The significant decline in catalytic efficiency of these three substrates was caused by lower substrate turnover numbers.

However, for the D-fuconate, the *K*_m_ value of H579L was more than threefold higher than that of the wild type (0.8 mM vs. 0.2 mM), and the *k*_cat_ value of H579L was about 20% higher than that of the wild-type enzyme (1290 min^−1^ vs. 1050 min^−1^). Consequently, the *k*_cat_/*K*_m_ of H579L was decreased compared with that of the wild-type enzyme (1570 mM^−1^ min^−1^ vs. 4400 mM^−1^ min^−1^), demonstrating a declined catalytic efficiency. The declined catalytic efficiency was due to the decrease in the affinity of the H579L variant for D-fuconate, which manifested as an increase in *K*_m_. A higher *K*_m_ indicates that the H579L variant requires more substrates to reach saturation. When the substrate is saturated, the reaction rate is the maximum rate, which is related to *k*_cat_. Therefore, in the case of substrate saturation, the variant's increased *k*_cat_ will cause it to exhibit higher catalytic activity. As shown above, the variant H579L had higher activity toward 10 mM D-fuconate than the wild-type one. We also measured the kinetic constants for D-galactonate, and the *K*_m_ value of the H579L variant was 0.9 mM, which was slightly higher than that of the wild-type enzyme (*K*_m_ = 0.7 mM). Meanwhile, the *k*_cat_ value dropped about eightfold for the H579L variant. Thus, the mutation of His to Leu at position 579 resulted in around a tenfold decrease in the catalytic efficiency of D-galactonate.

### Crystal structure of the H579L variant in complex with a product analog 2-oxobutyrate

To study the detailed effect of C-terminus mutation on the three-dimensional structure, the crystal structure of variant H579L with product analog 2-oxobutyrate was determined. To the best of our knowledge, this is the first available ligand complex structure of an enzyme from the IlvD/EDD superfamily. The crystal diffracted at 2.4 Å, and the structure was solved by molecular replacement. Four polypeptide chains, or a tetramer, were observed in the asymmetric unit. Superimposition of the structures of the H579L variant and the wild type of *Rl*ArDHT (holo form, PDB ID: 5J84) (Rahman et al. 2017) shows no major change in structure caused by the mutation, as indicated by the root mean square deviation (RMSD) value of atomic positions being 0.34 Å. Previously published structures of *Rl*ArDHT have shown that the enzyme has two forms, open or closed, because of conformational changes in the helix − loop − helix region (Gly166 − Ser192). This conformational alternation opens a tunnel into the active site that allows the substrate to approach. In the H579L variant structure, this helix − loop − helix region was observed in the closed-form conformation. The closed conformation may reduce the dissociation of [2Fe-2S] clusters, as indicated by the high occupancy values in the crystal structure (chain A, 0.73; chain B, 0.78; chain C, 0.80; chain D, 0.91).

**Fig. 3:**
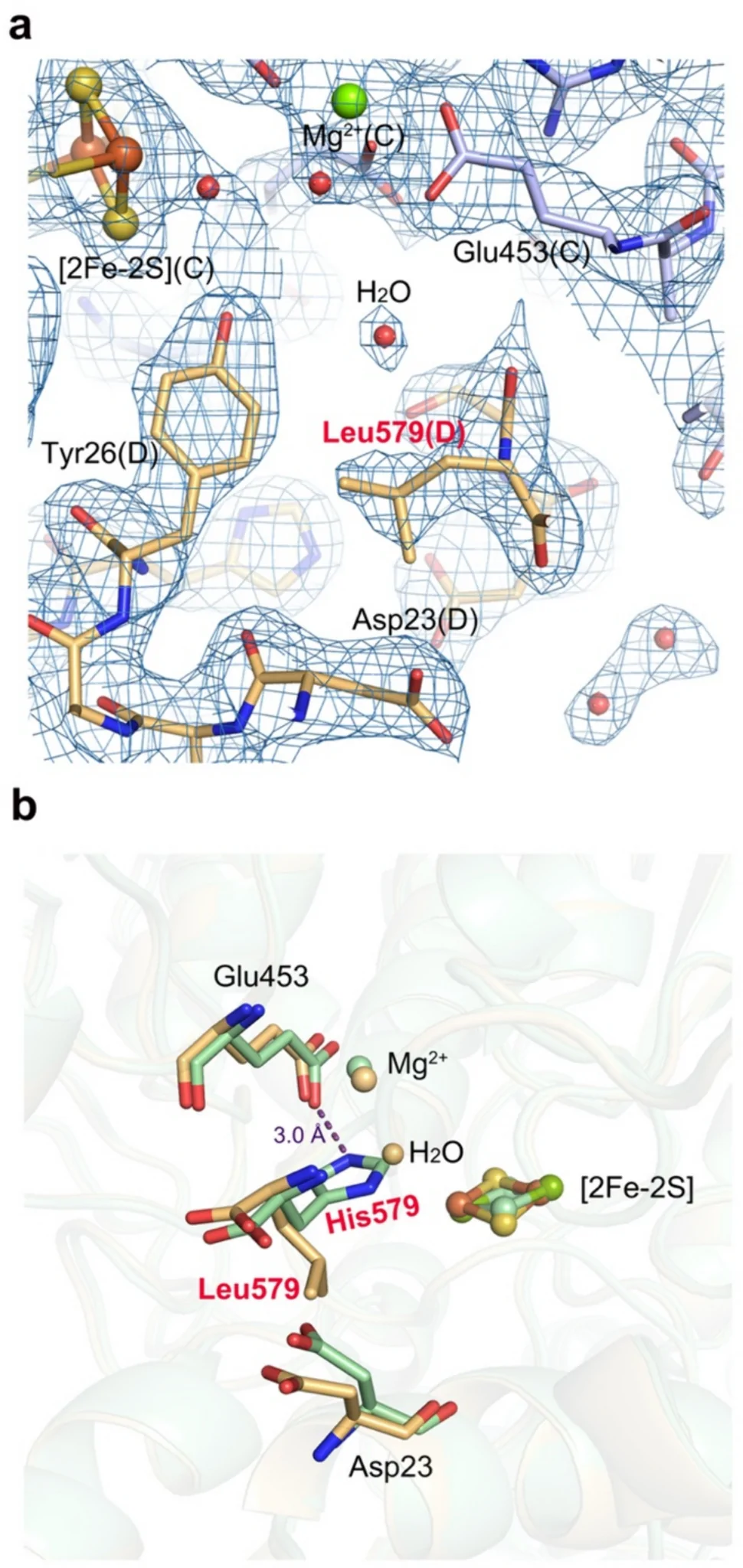
**a** 2F_o_-F_c_ electron density map of the C-terminus in the crystal structure of the H579L variant (PDB ID: 9EVV), contoured at the 1.0σ level. Chain C is shown in purple-blue, and chain D is shown in light orange. **b** Superimposition of the wild-type (in green) and the H579L variant (in light orange) structures. The side chain of Leu points away from the Mg^2+^ site, which allows for the binding of an extra water molecule into the cavity

The electron density map confirmed that there was a Leu instead of a His at the C-terminus in the H579L variant (Fig. 3a). Compared with the location of the side chain of His579 as seen in the wild type of *Rl*ArDHT, the side chain of Leu579 in the H579L variant showed a clearly distinct orientation (Fig. 3b). In the wild-type *Rl*ArDHT structure, the side chain of His579 is toward the active site, and its atom NE2 is 3.0 Å away from atom OE1 of Glu453 in the adjacent monomer. When the His residue is substituted for the Leu residue, the interaction with the Glu453 residue vanishes, and the side chain of Leu is rotated. The rotated Leu side chain also induces the conformational change of a nearby residue, Asp23, from the recognition helix. These subtle changes result in a larger substrate-binding cavity despite the almost interchangeable size of the residues His and Leu. An additional water molecule was present in the cavity originally occupied by the His side chain in polypeptide chain C. The enlarged substrate-binding cavity may facilitate substrate turnover, which was consistent with the approximately 20% increase in *k*_cat_ seen in the H579L variant compared with the wild type on the most preferred substrate D-fuconate.

**Fig. 4:**
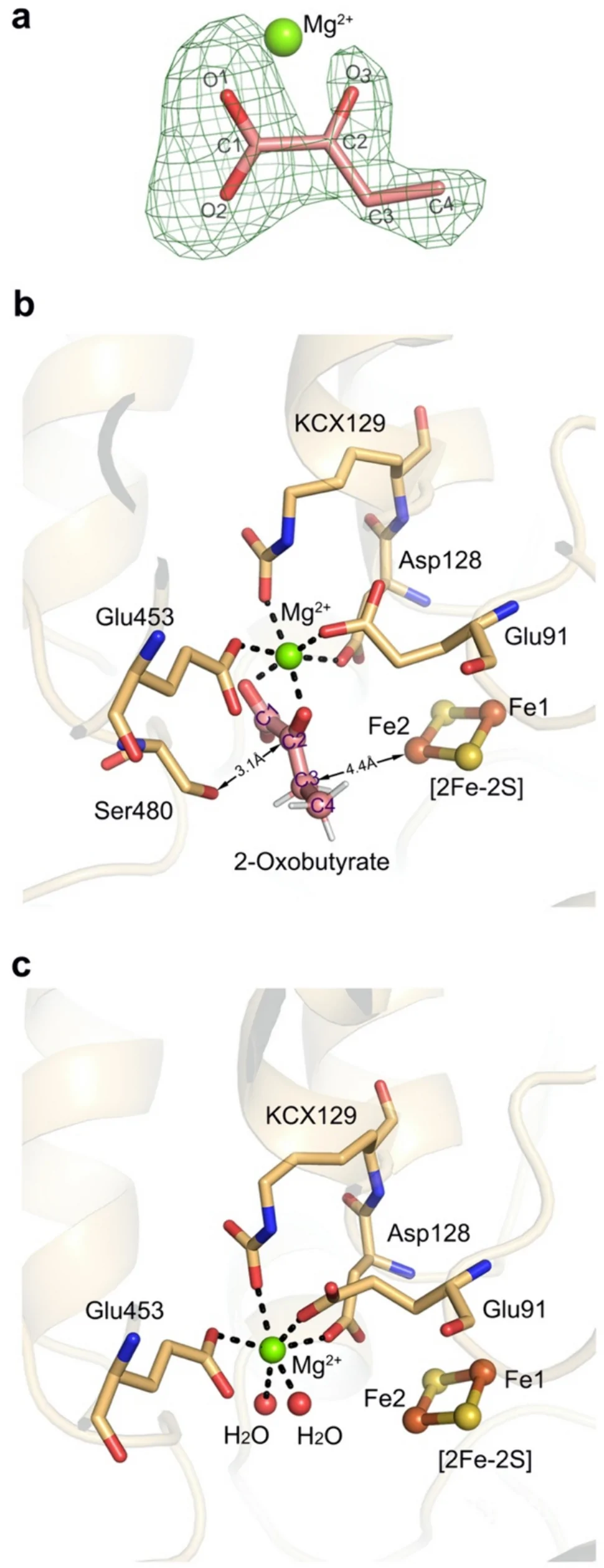
**a** Observed 2-oxobutyrate in molecule D of the H579L variant structure. The omit map for the 2-oxobutyrate is colored green, contoured at the 3.0σ level. The substrate-binding pocket in chain D (**b**) and in chain C (**c**) of the H579L variant

2-Oxobutyrate was refined inside the substrate-binding cavity in chain D of the H579L variant (Fig. 4a). A polder map that excludes the bulk solvent around the omitted region is shown in Supplemental Fig. S5. The oxygen atom O1 of the carboxylate group at C1 position and the oxygen atom O3 of the carbonyl group at C2 of 2-oxobutyrate were both coordinated to the Mg^2+^. In addition to the 2-oxobutyrate ligand, the residues Glu91, Asp128, KCX129 (6N-carboxylysine), and Glu453 were coordinated to Mg^2+^, leading to octahedral coordination geometry (Fig. 4b). In the three other chains, two water molecules replaced the two oxygen atoms of 2-oxobutyrate to participate in the octahedral coordination geometry (Fig. 4c).

*Rl*ArDHT catalyzes the elimination reaction of the hydroxyl group at position C3 and the proton at position C2 of the substrate to form a product with a keto group at the C2 position. The side chain of the Ser480 was oriented toward carbon C2 with a distance of 3.1 Å. The distance between C3 of 2-oxobutyrate and Fe2 of the [2Fe-2S] cluster was 4.4 Å, which would leave sufficient space for the hydroxyl group of the substrate.

In the three other chains, a water molecule occupied the corresponding space and formed a tetrahedral coordination geometry of Fe2 with two bridged sulfides and Cys200. Because 2-oxobutyrate has no hydroxyl group at position C4, contrary to the real substrate (or product), the crystal structure cannot clarify the stereospecificity of *Rl*ArDHT at the C4 position. Outside the carbon tail of 2-oxobutyrate, there is still enough space in the substrate-binding cavity, which allows accommodation of longer sugar acids with different lengths (C5 and C6) (Supplemental Fig. S6).

## Discussion

Sugar acid dehydratases are the bottleneck enzymes that catalyze the rate-limited reaction step, the conversion of sugar acid to 2-dehydro-3-deoxy sugar acid in microbial non-phosphorylative oxidative pathways. *Rl*ArDHT is an L-arabinonate dehydratase that belongs to the IlvD/EDD superfamily. The C-terminal histidine of *Rl*ArDHT participates in the formation of the active site of the adjacent monomer. The C-terminal histidine is fully conserved among members of the sugar acid dehydratase subfamily, but other IlvD/EDD enzymes have different structural arrangements. This suggests that the C-terminus might have a significant role in catalysis, particularly in substrate recognition.

Five variants (H579A, H579F, H579L, H579Q, and H579W) of the histidine residue at the C-terminus were established in this study. The activity on various C5/C6 sugar acids of these five variants was measured and compared to the wild type (Table 2). For D-galactonate, L-arabinonate, and D-xylonate, all five C-terminal variants had decreased activities, but for D-fuconate, which is the optimal substrate of *Rl*ArDHT, the variant H579L showed 20% higher activity than the wild type.

The size of the amino acid residue at the C-terminus is critical. The most dramatic decrease in activity was obtained when histidine was mutated to tryptophan. Although the indole ring of tryptophan and the imidazole ring of histidine both contain the NH group, the bulky side chain of tryptophan may interfere with access to the catalytic site. The obvious decreased activities of variants H579A and H579F further support the requirement on the size of the amino acid residue at the C-terminus. In variants H579Q and H579L, C-terminal histidine was substituted by amino acid residues of similar sizes, and the activities of these two variants on D-fuconate retained the same or a higher order of magnitude, respectively, compared to the wild type. The side chain size of the active site residue in 2,3-oxidosqualene cyclases from *Avena strigose* has been demonstrated to affect the deprotonation positions of the substrate (Liang et al. 2022).

The further kinetic analysis of the variant H579L showed that both the *Rl*ArDHT wild-type enzyme and the H579L variant had the same stereoselectivity for the S configuration at the C4 position of the substrate, shown as a significantly lower *k*_cat_/*K*_m_ on D-xylonate and D-gluconate than that on D-fuconate, L-arabinonate, and D-galactonate. The highest catalytic efficiency of the *Rl*ArDHT wild-type enzyme appeared to be on D-fuconate, followed by D-galactonate and L-arabinonate. However, the order of substrate preference for the H579L variant was D-fuconate, L-arabinonate, and D-galactonate. When the catalytic efficiency of the optimal substrate D-fuconate was taken as a reference, the relative catalytic efficiencies of the H579L variant for D-galactonate and L-arabinonate were 6% and 8%, respectively, and those of the wild type were 21% and 13%, respectively. Although the kinetic constants for the other variants were not determined, the relative activities of these variants for other C5/C6 substrates were much lower than those of the wild type. Andberg et al. (2016) have previously reported that the optimal substrate for wild-type *Rl*ArDHT is D-fuconate, but the catalytic efficiency toward L-arabinonate has been higher than toward D-galactonate. Here, a slightly different order of substrate preference for the wild-type enzyme could perhaps be attributed to the lower concentration of Mg^2+^ and the slightly different pH adopted in the reaction system.

The solved crystal structure of variant H579L showed an enlarged substrate-binding cavity, which may be the structural basis for the higher turnover of variant H579L on the D-fuconate substrate. The size of the substrate-binding cavity is known to be related to substrate specificity; for example, Ibba et al. (1994) have proved that the substrate specificity of an phenylalanyl-tRNA synthetase from *E.coli* was altered with an increase or decrease in the size of substrate-binding pocket. Sofeo et al. (2019) have expended the substrate range of an acetyl-CoA synthetase from *A. thaliana* by modifying the size and chemo-physical properties of its carboxylate binding pocket. Therefore, the alteration of the substrate specificity of variant H579L is most likely affected by the enlarged substrate-binding cavity.

Sutiono et al. (2020) discovered a sugar acid dehydratase from *P. ureilyticus* (*Pu*DHT). *Pu*DHT has functioned best on D-gluconate, followed by D-xylonate and L-threonate. However, it displayed minimal activity on D-glycerate, a C3 sugar acid. As mentioned previously, *Pu*DHT also contains a C-terminal histidine like *Rl*ArDHT. By substituting the native C-terminal histidine with a larger phenylalanine that altered the size of the substrate-binding cavity, Melse et al. (2022) increased the activity of *Pu*DHT on D-glycerate by sixfold and shifted its substrate preference from longer sugar acids toward shorter sugar acids. The catalytic variations of *Pu*DHT and *Rl*ArDHT caused by the mutation of its C-terminal histidine supported the hypothesis that the C-terminus of SADHTs might play an important role in the catalysis and that the size of the substrate-binding cavity of SADHTs affects the substrate specificity. In addition, Melse et al. have revealed other hotspots in *Pu*DHT that affect enzyme activity, for example, Glu89, Thr165, Gly202, and SGTA motif (476 − 479).

Furthermore, the solved crystal structure of variant H579L showed a 2-oxobutyrate at the substrate-binding cavity in chain D. This is the first complex structure of an enzyme from the IlvD/EDD superfamily. The complex structure supported our previously suggested hypothesis regarding the catalytic mechanism of *Rl*ArDHT, in which the residue Ser480 of *Rl*ArDHT has been proposed to be responsible for abstracting a proton from carbon C2 by its alkoxide side chain during the dehydration reaction. In addition, the Fe2 in the [2Fe-2S] cluster works as a Lewis acid that accepts the electron pair from the leaving hydroxyl group on C3. An alternative binding mode in which the carboxylate group of the ligand is bound to Fe2 has been proposed (Zhang et al. 2020), but this binding mode is not supported by our complex structure. The Ser642 in aconitase, an iron–sulfur enzyme that catalyzes the stereo-specific isomerization of citrate to isocitrate via cis-aconitate in the tricarboxylic acid cycle, is shown to abstract the proton from the C2 atom of the citrate substrate (Lloyd et al. 1999) and Fe^3+^ in [Fe-S] is known to act as a Lewis acid to activate the hydroxyl group (Emptage et al. 1983; Flint and Allen 1996).

In conclusion, our results revealed a significant functional role for the last C-terminal histidine residue of *Rl*ArDHT. Mutation of the histidine changed the catalytic activity of *Rl*ArDHT. The detailed kinetic analysis indicated that the H579L variant improved the substrate preference toward D-fuconate. The solved crystal structure of the H579L variant showed that the substitution of histidine by leucine enlarged the substrate-binding cavity. The refined product analog 2-oxobutyrate in chain D further supported the reaction hypothesis. This study demonstrates that the alteration of the substrate specificity of sugar acid dehydratases is possible, which offers opportunities in enzyme and metabolism engineering.

## Supplementary Information

Below is the link to the electronic supplementary material.

Supplementary file1 (PDF 5472 KB)

## Data Availability

The coordinate file and the structure factor file for the crystal structure of the H579L variant have been deposited in the Protein Data Bank with the accession code 9EVV.
