# Supplementary material for: Unveiling the importance of the C-terminus in the sugar acid dehydratase of the IlvD/EDD superfamily
Source: Appl Microbiol Biotechnol. 2024 Aug 10;108(1):436. doi: 10.1007/s00253-024-13270-8 (PMC11316719; doi:10.1007/s00253-024-13270-8)
Supplement: Supplementary file 1 — Supplementary file1 (PDF 5472 KB) [file 253_2024_13270_MOESM1_ESM.pdf]

Applied Microbiology and Biotechnology

Supplemental Material

Unveiling the importance of the C-terminus in the sugar acid dehydratase of the IlvD/EDD superfamily

Yaxin Ren [1], Elias Vettenranta [1], Leena Penttinen [1], Martina Andberg [2], Anu Koivula [2], Juha Rouvinen [1], Nina Hakulinen [1, *]

[1] Department of Chemistry, University of Eastern Finland, 111, 80101 Joensuu, Finland.

[2] VTT Technical Research Centre of Finland Ltd, Espoo, Finland.

[*] Corresponding author:

Nina Hakulinen: nina.hakulinen@uef.fi

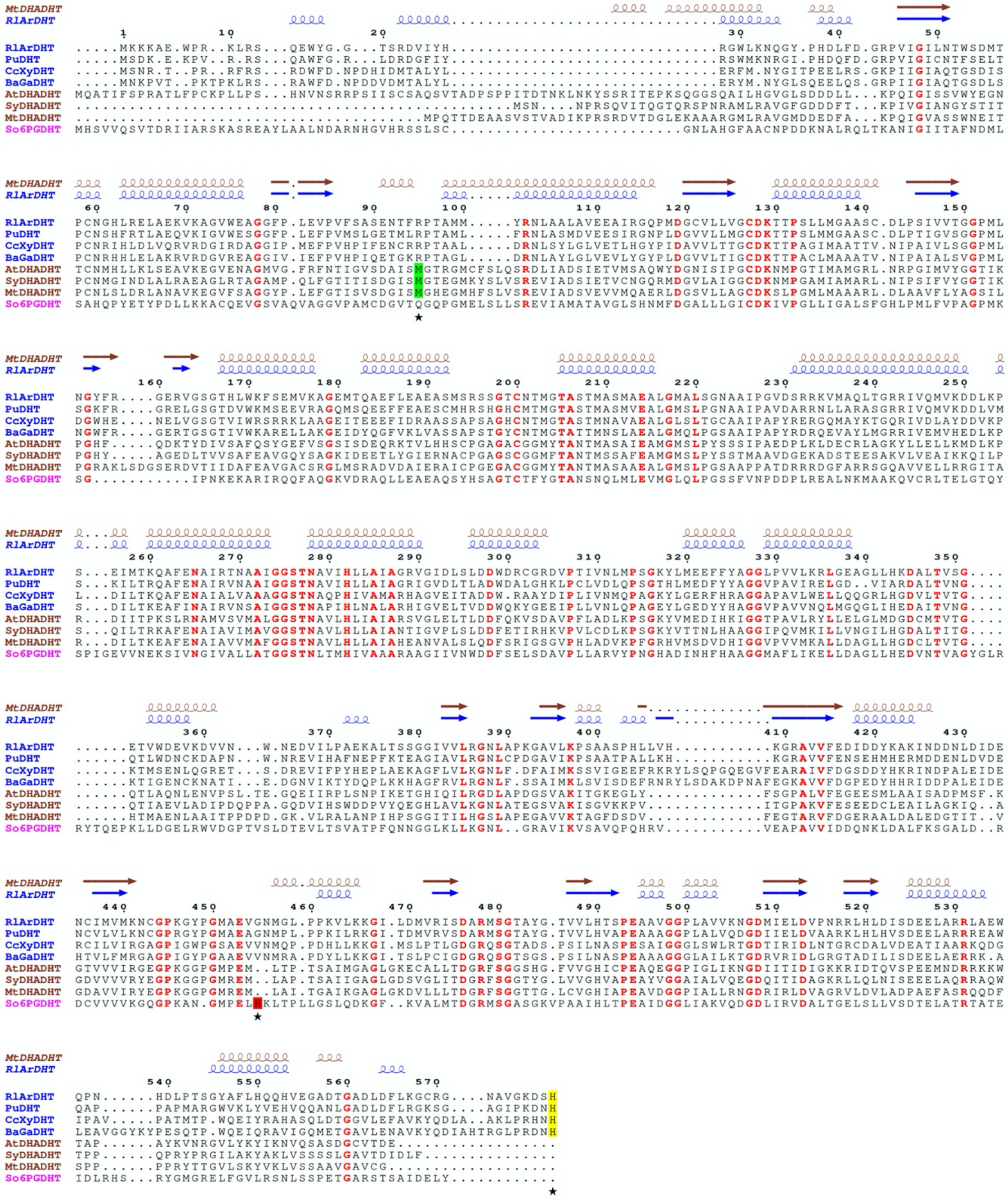

Figure S1 Full multiple sequence alignment of all IlvD/EDD superfamily dehydratases with the known crystal structures. *Rl*ArDHT is an L-arabinonate dehydratase from *Rhizobium leguminosarum* bv. *trifolii* (PDB ID: 5J84); *Pu*DHT is a sugar acid dehydratase from *Paralcaligenes ureilyticus* (PDB ID: 8EPZ); *Cc*XyDHT is a D-xylonate dehydratase from *Caulobacter crescentus* (PDB ID: 5OYN); *Ba*GaDHT is a galactonate dehydratase from *Brucella abortus* 2308 (PDB ID: 7M3K); *At*DHADHT is a dihydroxy acid dehydratase from *Arabidopsis*

thaliana (PDB ID: 5YM0); *Sy*DHADHT is a dihydroxy acid dehydratase from *Synechocystis* sp. PCC 6803 (PDB ID: 6NTE); *Mt*DHADHT is a dihydroxy acid dehydratase from *Mycobacterium tuberculosis* (PDB ID: 6OVT); and *So*6PGDHT is a 6-phosphogluconate dehydratase from *Shewanella oneidensis* MR-1 (PDB ID: 2GP4). The names of the sugar acid dehydratases are shown in blue, and those of the branched chain acid dehydratases are in brown. The conserved C-terminal histidine residues of sugar acid dehydratases are marked in yellow, the conserved methionine residues in branched chain acid dehydratases are marked in green, and the His488 residue in *So*6PGDHT is marked in red. These are also marked with a black star at the bottom of the sequences. Fully conserved residues are represented in red. The numbers above the sequences indicate the residue number of *Rl*ArDHT. The secondary structure elements of *Mt*DHADHT and *Rl*ArDHT are also shown

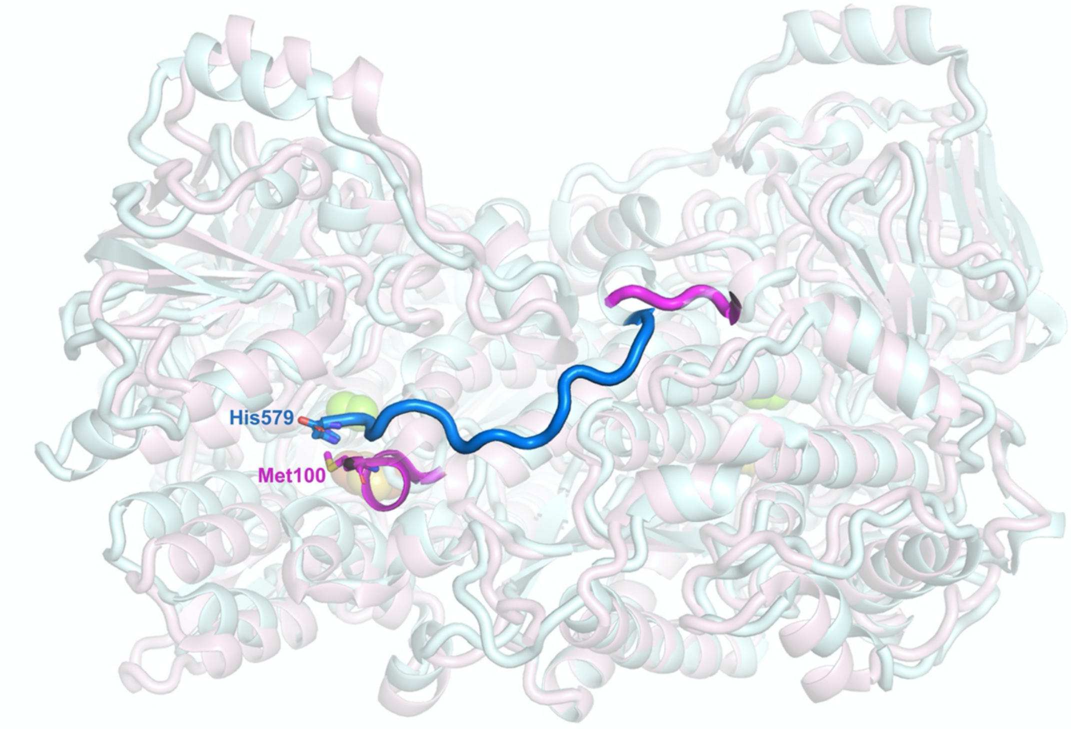

Figure S2 Superimposition between the dimeric unit of L-arabinonate dehydratase from *Rhizobium leguminosarum* bv. *trifolii* (*Rl*ArDHT, PDB ID: 5J84, in blue) and the dimeric unit of dihydroxy acid dehydratase from *Mycobacterium tuberculosis* (*Mt*DHADHT, PDB ID: 6OVT, in pink). The long C-terminal tail from *Rl*ArDHT is in deep blue. The short C-terminal tail and the short extra α -helix from *Mt*DHADHT is in magenta

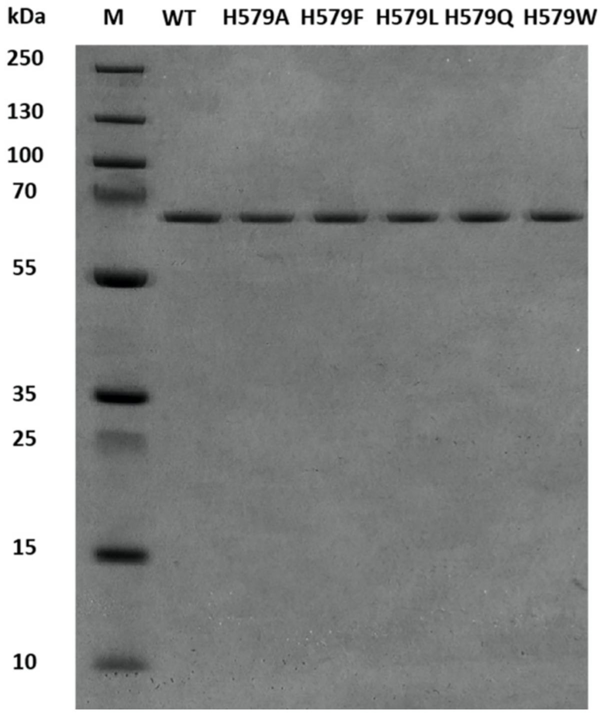

Figure S3 SDS-PAGE analysis of purified wild-type recombinant *Rl*ArDHT (marked as WT) and its five C-terminal variants (marked as H579A, H579F, H579L, H579Q, and H579W). The molecular mass markers (in kDa) are labeled on the left in lane M

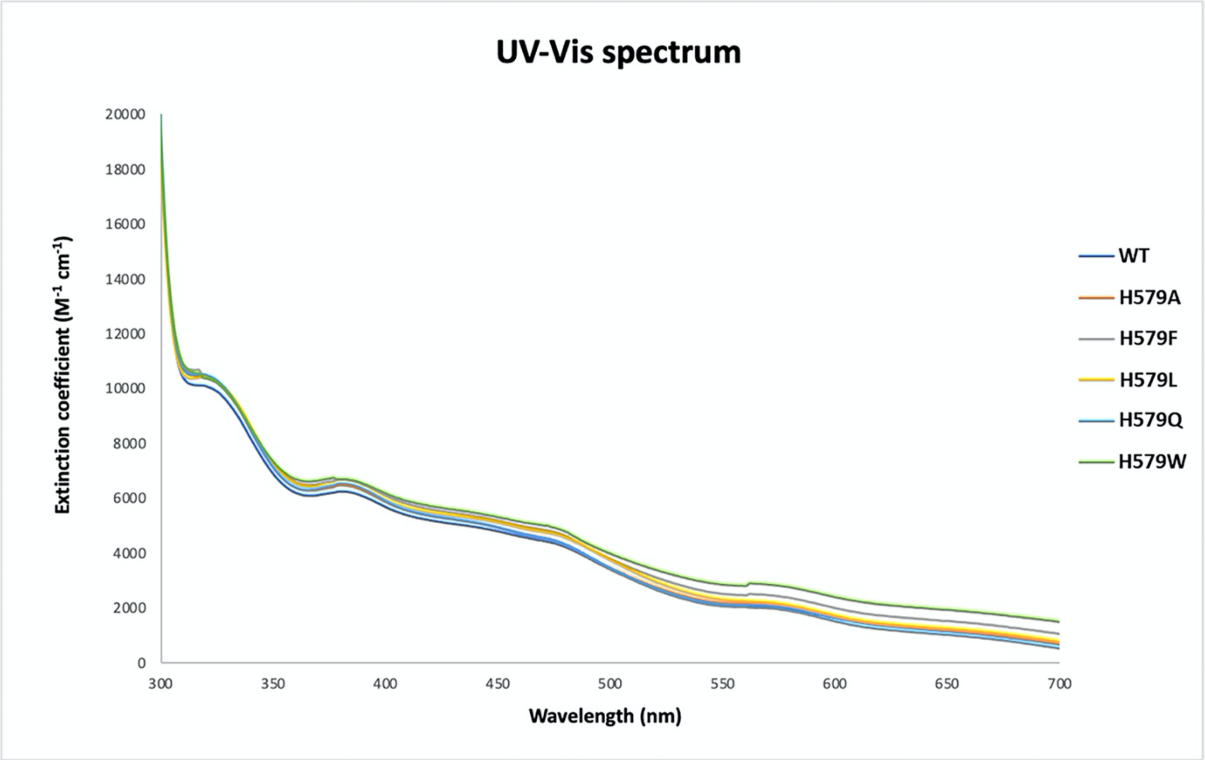

Figure S4 Ultraviolet−visible spectra (300−700 nm) for the wild-type *Rl*ArDHT (in blue) and its C-terminal variants

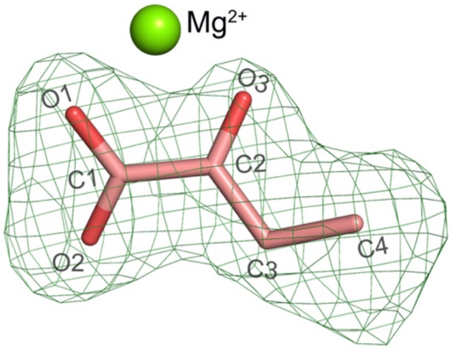

Figure S5 A polder map (contoured at the 6.0σ level) for a 2-oxobutyrate in molecule D of the H579L variant

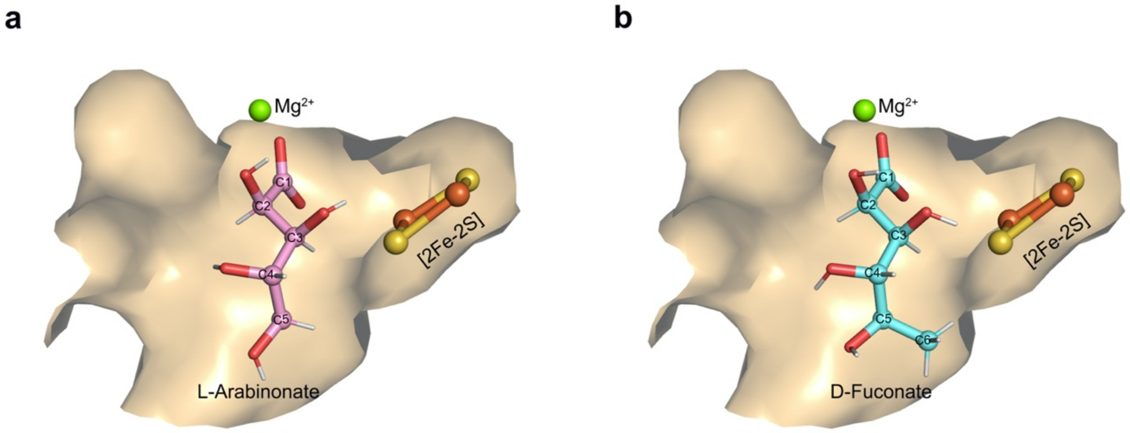

Figure S6 Substrate-binding cavity of the H579L variant docked with L-arabinonate (a) and D-fuconate (b) to visualize that there is sufficient space in the substrate-binding cavity to accommodate longer sugar acids (C5 and C6)
